# Reprogramming of VEGF-mediated extracellular matrix changes through autocrine signaling

**DOI:** 10.1080/15384047.2023.2184145

**Published:** 2023-03-09

**Authors:** Eibhlin Goggins, Yelena Mironchik, Samata Kakkad, Desmond Jacob, Flonne Wildes, Zaver M. Bhujwalla, Balaji Krishnamachary

**Affiliations:** a Division of Cancer Imaging Research, The Russell H. Morgan Department of Radiology and Radiological Science, The Johns Hopkins University School of Medicine, Baltimore, MD, USA; b Sidney Kimmel Comprehensive Cancer Center, The Johns Hopkins University School of Medicine, Baltimore, MD, USA; c Department of Radiation Oncology and Molecular Radiation Sciences, The Johns Hopkins University School of Medicine, Baltimore, MD, USA

**Keywords:** VEGF, TNBC, ECM, collagen, fibronectin, hyaluronan, degradative enzymes, CAFs, uPAR, autocrine signaling

## Abstract

Vascular endothelial growth factor (VEGF) plays key roles in angiogenesis, vasculogenesis, and wound healing. In cancers, including triple negative breast cancer (TNBC), VEGF has been associated with increased invasion and metastasis, processes that require cancer cells to traverse through the extracellular matrix (ECM) and establish angiogenesis at distant sites. To further understand the role of VEGF in modifying the ECM, we characterized VEGF-mediated changes in the ECM of tumors derived from TNBC MDA-MB-231 cells engineered to overexpress VEGF. We established that increased VEGF expression by these cells resulted in tumors with reduced collagen 1 (Col1) fibers, fibronectin, and hyaluronan. Molecular characterization of tumors identified an increase of MMP1, uPAR, and LOX, and a decrease of MMP2, and ADAMTS1. α-SMA, a marker of cancer associated fibroblasts (CAFs), increased, and FAP-α, a marker of a subset of CAFs associated with immune suppression, decreased with VEGF overexpression. Analysis of human data from The Cancer Genome Atlas Program confirmed mRNA differences for several molecules when comparing TNBC with high and low VEGF expression. We additionally characterized enzymatic changes induced by VEGF overexpression in three different cancer cell lines that clearly identified autocrine-mediated changes, specifically uPAR, in these enzymes. Unlike the increase of Col1 fibers and fibronectin mediated by VEGF during wound healing, in the TNBC model, VEGF significantly reduced key protein components of the ECM. These results further expand our understanding of the role of VEGF in cancer progression and identify potential ECM-related targets to disrupt this progression.

## Introduction

Vascular endothelial growth factor-A (VEGF-A or VEGF) is a versatile dimeric glycoprotein that plays important roles in normal tissue function such as in wound-healing and embryonic development, and in pathologies such as diabetic and hypersensitive retinopathy, rheumatoid arthritis, age-related macular degeneration, and cancer.^1^ Factors that regulate VEGF include hypoxia inducible factor-1α (HIF1-α), nuclear factor kappa-B (NF-kB), transforming growth factor (TFG-β), endothelin-1 and mechanical stress.^2,3^ VEGF is a potent angiogenic and vascular permeability factor and its expression is tightly coupled to oxygenation due to the presence of several hypoxia response elements in its promoter region.^4^ In cancers, VEGF plays an important role in tumor angiogenesis, vascular permeability, tumor growth, and metastasis.^1^ VEGF has six main isoforms, VEGF111, VEGF121, VEGF145, VEGF165, VEGF189, and VEGF206^5–7^ that have distinct effects on tumor growth and progression.^8^ Of these, VEGF165 has been extensively studied as it is the most frequently expressed isoform in tissues.^1^

The role of VEGF signaling in cancer, beyond its role in angiogenesis, is rapidly evolving. VEGF promotes cancer cell proliferation, migration and invasiveness,^9^ promotes stemness,^10,11^ and promotes immune suppression.^12^ Increased VEGF expression has been identified in several cancers,^13^ and it is associated with poor prognosis and increased metastasis in multiple cancers including triple negative breast cancer (TNBC).^14–18^ The VEGF targeted monoclonal antibody, bevacizumab, is approved in a range of solid tumor indications.^19^

Because of the role of VEGF in tumor angiogenesis as well as in invasion and metastasis, there is significant interest in understanding the role of VEGF in modifying the tumor extracellular matrix (ECM). Cancer cells have to navigate through the ECM on their metastatic journey^20^ and the establishment of neovasculature requires ECM remodeling.^20,21^ We previously identified increased matrigel degradation by VEGF overexpressing MCF-7 human breast cancer cells in an intact-cell perfusion system.^9^ *In vivo* magnetic resonance imaging (MRI) studies with VEGF overexpressing MCF-7 and MDA-MB-231 tumors, revealed a significant increase of vascular volume and permeability, changes in macromolecular transport through the ECM, and increased metastasis.^9^

Studies investigating the effects of VEGF on the tumor ECM have primarily focused on characterizing changes in the matrix metalloproteinases (MMPs) and other degradative enzymes.^22,23^ Effects of increased VEGF expression or VEGF targeting with bevacizumab on bone cartilage and osteoarthritis have also been previously described.^24^ Here, we have directly characterized changes in key ECM components such as collagen 1 (Col1), fibronectin (FN1), and hyaluronan (HA), together with alterations in degradative enzymes and cancer associated fibroblast (CAF) markers, to understand how VEGF alters the ECM in a human TNBC xenograft overexpressing VEGF165. We independently confirmed degradative enzyme changes in human TNBC with high and low VEGF mRNA levels by analyzing data from The Cancer Genome Atlas Program. We additionally characterized enzymatic changes in VEGF overexpressing MDA-MB-231 cells, as well as in human prostate cancer PC-3 cells, and in estrogen receptor (ER) positive MCF-7 breast cancer cells engineered to overexpress VEGF. Several of the changes in MDA-MB-231 VEGF overexpressing tumors were also observed in the cells suggesting that autocrine signaling mediated these changes. A significant increase of uPAR was observed with VEGF overexpression in both MDA-MB-231 and PC-3 cells, but not in MCF-7 cells. These results expand our understanding of the role of VEGF in ECM remodeling and provide new insights into the role of VEGF in TNBC.

## Materials and methods

### Cells and tumors

MDA-MB-231, PC-3 and MCF-7 cancer cells were obtained from ATCC (Manassas, VA). Establishment of VEGF165 overexpressing cancer cells was done as previously described^9^ and validated for VEGF expression by ELISA following manufacturer’s instruction (R&D, Minneapolis, MN)^9,25^ and by RT-PCR.

Two million MDA-MB-231 wild type (231_WT) or VEGF overexpressing (231_VEGF) cells were inoculated in the mammary fat pad of 4–6 weeks old female severe combined immunodeficient (SCID) mice. Tumors were excised once they reached a volume of ~300-500 mm^3^. Studies were performed with 5–10 tumors from each group. One half of each tumor was fixed in formalin for immunohistochemistry and the other half freeze-clamped for molecular analysis. 231_VEGF tumors were validated for VEGF expression by ELISA following manufacturer’s instructions (R&D, Minneapolis, MN).^9,25^ Animal handling was conducted in accordance with the regulations outlined by the Institutional Animal Care and Use Committee of Johns Hopkins University.

### Second harmonic generation (SHG) microscopy

SHG microscopy of hematoxylin and eosin (H&E) stained sections was performed as previously described.^26^ Briefly, tumors were paraffin-embedded and 5 μm thick H&E sections were used for SHG microscopy. Slides were analyzed using an Olympus Laser Scanning FV1000 MPE multiphoton microscope (Olympus Corp., US headquarters–Center Valley, PA) with a 25Xw/ 1.05XLPLN MP lens. Excitation was achieved at 860 nm and the second harmonic signal was detected at a wavelength of 430 nm. Col1 fiber parameters of percent fiber volume and inter-fiber distance were quantified, and Haralick texture features such as contrast and homogeneity were analyzed, using an in-house fiber analysis software written in MATLAB 7.4.0 (The MathWorks, Natick, MA, USA) as previously described.^26^

### Immunohistochemistry

Formalin-fixed, paraffin-embedded sections of tumors were deparaffinized followed by antigen retrieval. Antibodies used for immunohistochemistry (IHC) of targets-of-interest were: rat monoclonal CD31 antibody (Dianova, Hamburg, Germany) at 1:30 dilution, rabbit anti-Col1A1 antibody cross-reactive with mouse and human Col1A1 (OriGene, Rockville, MD, USA) at 1:70 dilution, mouse-monoclonal anti-FN1 antibody cross-reactive with mouse and human FN1 (Immunogen- Fusion proteinAg8016, Proteintech, Rosemont, IL, USA) at 1:100 dilution, and bovine nasal cartilage HABP (Millipore Sigma, Merck KGaA, Darmstadt, Germany) at 1:750 dilution. Slides were incubated overnight at 4°C. Following this, sections were incubated with horseradish peroxidase conjugated with anti-mouse or anti-rabbit IgG. For HABP, the VECTASTAIN ABC-AP Kit procedure was used. Finally, slides were stained with 3,3′-diaminobenzidine (DAB) and counterstained with hematoxylin.

High-resolution digital scans of the stained sections (five tumors per group, 1 section analyzed per tumor) were obtained using ScanScope (Aperio, Vista, CA). Quantification was done with the ImageScope software using the Positive Pixel Count V9 algorithm supplied by the manufacturer. The number of strongly positive or positive pixels normalized to the total number of pixels was obtained. Analyses were performed using the entire histological section, with entire viable and necrotic regions in the section mapped from adjacent H&E stained slides.

### RT-PCR and immunoblotting

RNA isolation was done using Qiagen kit (Qiagen, Valencia, CA, USA). To obtain RNA, tissues were homogenized with RLT buffer and passed through a QIAshredder. cDNA was synthesized using an iScript cDNA synthesis kit (Bio-Rad, Hercules, CA, USA).

For quantitative real-time PCR (qRT-PCR), 1 μl of 1:10 diluted cDNA was used. IQ SYBR Green Supermix and gene-specific primers in the iCycler RT-PCR detection system (Bio-Rad, Hercules, CA, USA) were used. For the ECM proteins, Col1A1, Col1A2, and FN1, and for fibroblast activation protein alpha (FAP-α), mouse ECM specific primers were designed. The house-keeping genes, hypoxanthine phosphoribosyltransferase-1 (HPRT-1) and 18s ribosomal RNA (18s rRNA), were used as controls. The threshold cycle (ct) from these house-keeping genes was used to calculate the expression of human and mouse-specific genes. The change in threshold cycle (Δct) values between HPRT-1 for targets of human origin and 18s for mouse related targets, and the gene of interest was calculated for 231_WT and 231_VEGF samples. To obtain ΔΔct and the fold mRNA expression, the average Δct of the 231_WT samples was subtracted from the Δct values of the 231_VEGF sample. Using the formula 2^−ΔΔct^, fold mRNA expression of individual samples was determined and plotted using GraphPad prism.

Protein isolation and immunoblotting was performed as previously described.^27^ Antibodies cross-reactive with mouse/human ECM proteins and specific for human enzymes of interest included rabbit-polyclonal anti-Col1A1 antibody (1:1000; OriGene, Rockville, MD, USA), mouse monoclonal anti-FN1 antibody (1:2000 dilution; Proteintech, Rosemont, IL, USA), rabbit polyclonal anti-MMP-1 antibody (1:1000 dilution; Neo BioLab, Woburn, MA, USA), rabbit polyclonal anti-MMP2 (1:1000 dilution; GeneTex, Inc., Irvine, CA, USA), rabbit polyclonal anti-MMP-14 antibody (1:1000 dilution; Neo BioLab, Woburn, MA, USA), mouse monoclonal anti-lysyl oxidase (LOX) antibody (1:1000 dilution; GeneTex, Inc., Irvine, CA, USA), mouse monoclonal anti-ADAMTS1 antibody (1:500 dilution; OriGene, Rockville, MD, USA), rabbit polyclonal anti-uPAR (1:1000 dilution; GeneTex, Inc., Irvine, CA, USA), mouse monoclonal anti-α-SMA antibody (1:2000; Novus Biologicals, Littleton, CO, USA), rabbit monoclonal antibody against neuropilin-1 (NRP-1) (1:1000, clone D62C6, Cell Signaling, Danvers, MA, USA, rabbit polyclone anti-FLT1 (VEGFR1) antibody (1:1000, MyBioSource, San Diego, CA) and rabbit polyclonal anti-FAP-α antibody (1:1000, Ab207178, Abcam, Cambridge, UK). Horseradish peroxidase-conjugated secondary antibodies were used at 1:2000 dilution. Blots were visualized using the SuperSignal West Pico Chemiluminescent substrate kit (Thermo Scientific, Rockford, IL, USA). The reference band from the molecular weight marker was used to determine the location of the protein of interest. Autoradiographs were scanned, and densitometry of the band intensities of various proteins of interest were obtained using ImageJ software. The band intensity for each protein was normalized to the intensity of GAPDH protein used as a loading control. Values are represented as Mean ± Standard Error of the Mean (SEM) from five individual tumor samples for the *in vivo* studies and at least three biological replicates for the cell studies.

### Human breast cancer analysis

Publicly available TCGA data sets for breast cancer were retrieved from the TCGA Data portal (https://tcga-data.nci.nih.gov/tcga)^28^ using the open-access, open-source, web-based platform cBioPortal for Cancer genomics (cbioportal.org).^29^ Clinical identifiers were applied to select treatment naïve patient samples that were triple negative. Three studies, the Korean breast cancer cohort study (SMC-2018),^30^ the breast invasive carcinoma TCGA study (TCGA-2015),^31^ and the breast invasive carcinoma TCGA Firehose Legacy study (Firehose Legacy), with source data from the repository at Broad Institute Genomic Data Analysis Center (GDAC), met our criteria. Based on curated RNA sequencing data for a given study, we next applied genomic filters to group patient samples with high and low VEGFA mRNA expression based on the z-score of samples (log RNA Seq V2 RSEM). Z-score values greater than 1.2-fold were grouped as VEGFA-high and values less than 1.2-fold were grouped as VEGFA-low. In the case of the TCGA study,^30^ RNA sequencing data were provided as transcripts per million (TPM). For this study, TPM values for VEGA expression were divided into four quartiles using the tool available in cBioPortal web portal. Data from the highest and lowest quartile were analyzed as high and low VEGFA expressing tumors. Comparison analyses for MMP1, uPAR, LOX and α-SMA were performed for VEGF-high and VEGF-low data sets following the instructions in cBioportal.^32^ The cBioportal-derived expression data presented in this study are based on a frequently employed analysis technique called the RNA-seq by Expectation Maximization (RSEM). RSEM takes into account the transcript length and provides acceptable and accurate results.^33^

### Statistical analysis

Statistical analysis was performed using GraphPad Prism (San Diego, CA). P values ≤ .05 were considered significant. P values were based on a two-tailed t-test for the ELISA and mRNA analysis, and a one-tailed t-test for the IHC studies, based on the sample size. For the TCGA data sets, a Mann-Whitney test was performed, as the non-parametric Mann-Whitney test is most appropriate for large-scale RNA seq data. Additionally, the TCGA data sets were also evaluated with a two-tailed t-test.

## Results

### Validation of VEGF overexpression in cells and tumors and its effects on tumor vasculature

**Figure 1.**
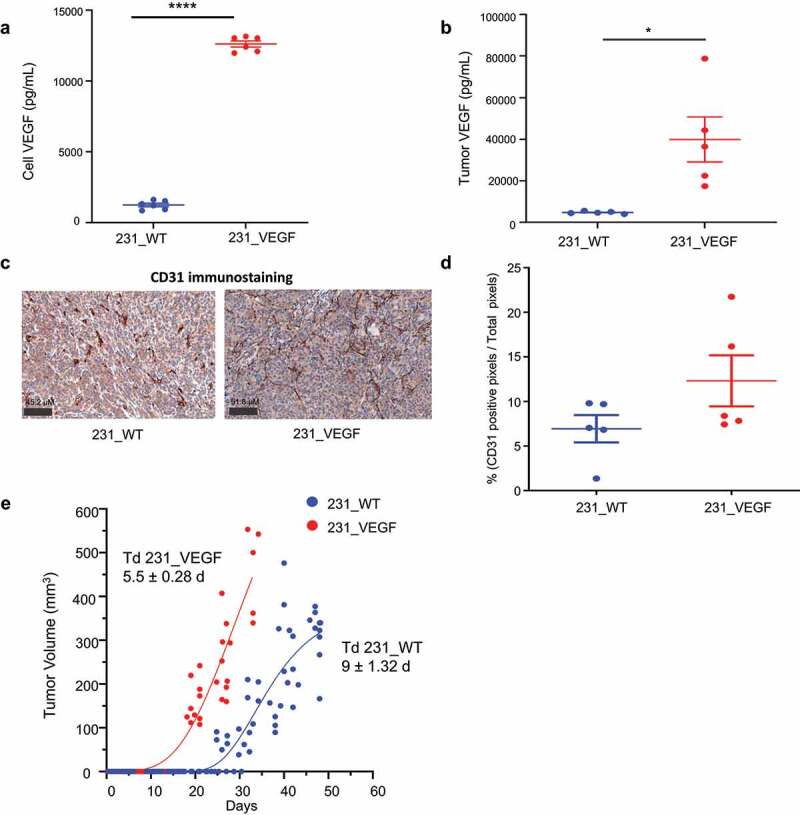
Validation of VEGF Overexpression. (a) Validation of the overexpression of VEGF in the supernatant of 231_VEGF cells (N = 5) compared to 231_WT cells (N = 5). (b) Validation of the overexpression of VEGF in tumors derived from 231_VEGF cells (N = 5) compared to tumors from 231_WT cells (N = 5). (c) Representative CD31 immunostained images from 231_WT (left) and 231_VEGF (right) tumors. (d) Quantification of strongly positive pixel (NSP) area normalized to the total pixel area in 231_WT (N = 5) and 231_VEGF (N = 5) tumors. 1 section was analyzed from each tumor. (e) Growth curves of 231_WT and 231_VEGF tumors (N = 10 per group); each point in the graph represents a single tumor volume. Values represent Mean ± S.E.M. *P ≤ .05,****P ≤ .00005.

ELISA performed on supernatant derived from cells and protein isolated from tumor-derived samples showed a statistically significant increase of VEGF in 231_VEGF cells (Figure 1a) and 231_VEGF tumors (Figure 1b) compared to wild-type cells and tumors. We used CD31 as a marker of endothelial cells to confirm the functional effects of VEGF on increasing tumor vasculature. As shown in the representative IHC images in Figure 1c,a higher number of CD31 immunostained pixels was detected in 231_VEGF tumors (right) compared to 231_WT tumors (left). These results summarized in Figure 1d identified a trend (P ≤ .07) of increased vessel density in VEGF overexpressing tumors. Growth curves for 231_WT and 231_VEGF tumors, fitted to a Gompertzian curve, are shown in Figure 1e. Tumor growth was significantly higher in 231_VEGF tumors compared to 231_WT tumors. A significant difference between the average doubling time for 231_WT and 231_VEGF tumors was observed (P ≤ .05). The tumor doubling time (Td) was approximately 9 ± 1.32 d for 231_WT tumors compared to 5.5 ± 0.28 d for 231_VEGF tumors, estimated for tumor volumes from 110 mm^3^ to 300 mm^3^ (values represent Mean ± S.E.M.). We additionally characterized VEGF mRNA as well as VEGF levels in cell supernatant and lysate by ELISA for PC-3 and MCF-7 cells overexpressing VEGF to confirm increased levels of VEGF in these cells as shown in Supplementary Figures 1a,b.

### VEGF overexpression reduced Col1, fibronectin, and hyaluronan

**Figure 2.**
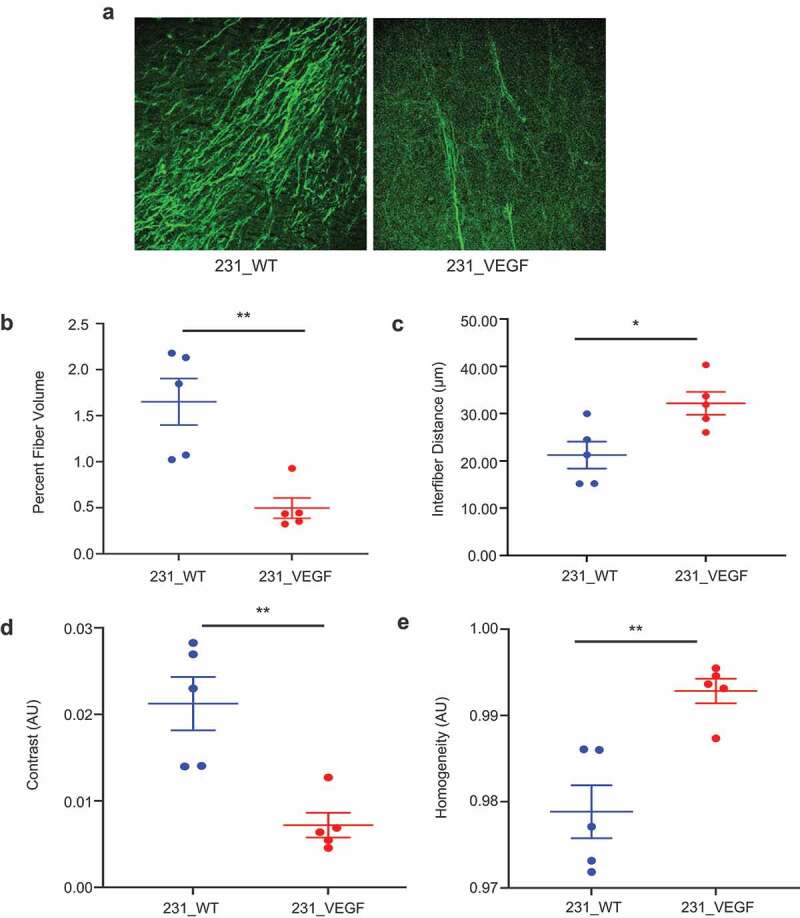
Col1 fiber changes detected with SHG microscopy. (a) Representative Col1 fiber images acquired using SHG microscopy from 231_WT (left) and 231_VEGF (right) tumors. The images were acquired at a pixel resolution of 0.83 μm x 0.83 μm with a FOV of 425 × 425 μm^2^.(b) Quantification of percent fiber volume, (c) Interfiber distance, (d) contrast and (e) homogeneity from SHG image analysis of 231_WT (N = 5) and 231_VEGF (N = 5) tumors. Values represent Mean ± S.E.M. * P ≤ .05, ** P ≤ .005.

We next characterized Col1 fibers in tumor sections using SHG microscopy. Representative images of Col1 fibers obtained using SHG microscopy in Figure 2a from 231_VEGF (right) and 231_WT tumors (left) show the significant decrease of Col1 fibers in VEGF overexpressing tumors. Compared to 231_WT tumors, percent fiber volume in 231_VEGF tumors significantly decreased (Figure 2b) and interfiber distance significantly increased (Figure 2c). Haralick feature analysis identified a significant decrease in contrast (Figure 2d) and a significant increase in homogeneity (Figure 2e) with VEGF overexpression.

**Figure 3.**
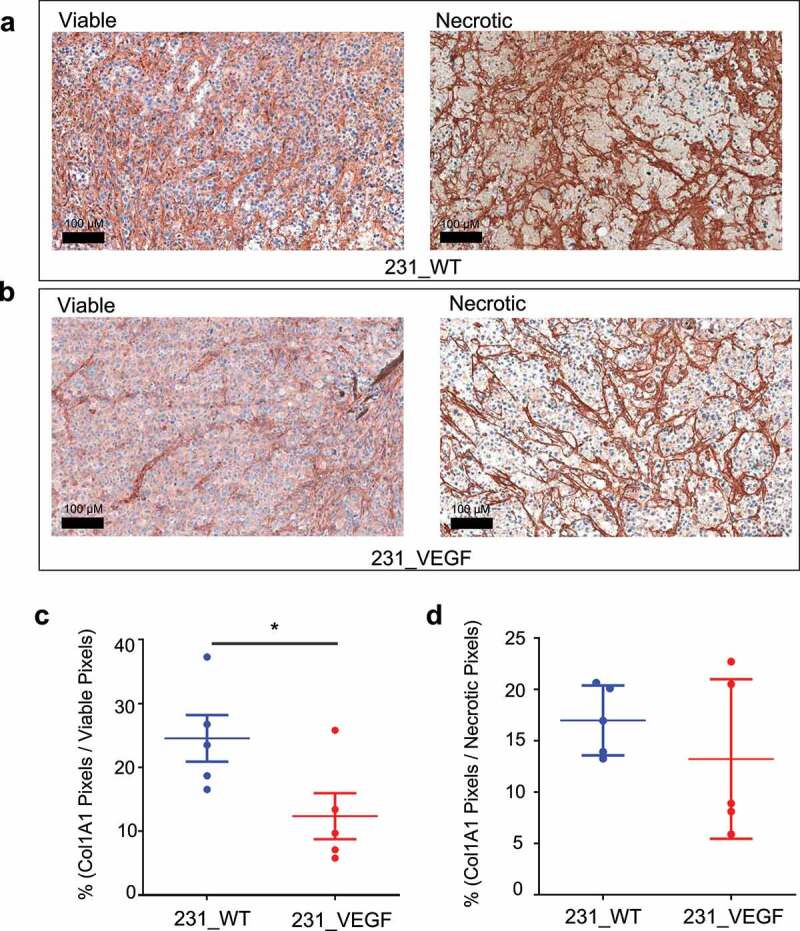
Col1A1 immunostaining. (a) Representative images of Col1A1 immunostained tumor sections from viable (left) and necrotic (right) regions of 231_WT tumors. (b) Representative images of Col1A1 immunostained tumor sections from viable (left) and necrotic (right) regions of 231_VEGF tumors. Quantification of Col1A1 positive pixels normalized to the total pixel area of (c) viable tumor regions and (d) necrotic tumor regions from 231_WT (N = 5) and 231_VEGF (N = 5) tumors. Values represent Mean ± S.E.M. * P ≤ .05.

**Figure 4.**
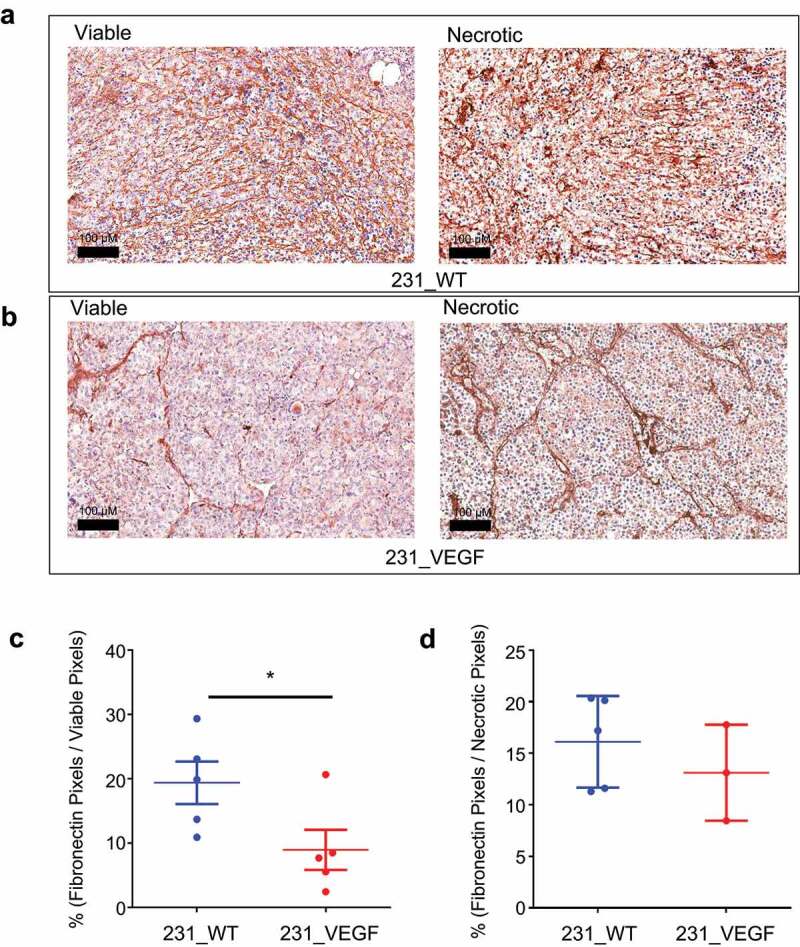
Fibronectin immunostaining. (a) Representative images of fibronectin immunostained tumor sections from viable (left) and necrotic (right) regions of 231_WT tumors. (b) Representative images of fibronectin immunostained tumor sections from viable (left) and necrotic (right) regions of 231_VEGF tumors. Quantification of fibronectin positive pixels normalized to the total pixel area of (c) viable tumor regions and (d) necrotic tumor regions from 231_WT (N = 5) and 231_VEGF (N = 5) tumors. Values represent Mean ± S.E.M. * P ≤ .05.

The significant decrease in Col1 fibers identified with SHG microscopy was further confirmed with IHC of Col1A1. Representative viable and necrotic areas of immunostained sections from 231_WT tumors in Figure 3a and 231_VEGF tumors in Figure 3b show decreased Col1A1 immunostaining in viable tumor regions of VEGF overexpressing tumors, but not in necrotic tumor regions. These data, summarized in Figure 3c,d, demonstrate the significant decrease of Col1A1 with VEGF overexpression in viable tumor regions (Figure 3c), but not in necrotic tumor regions (Figure 3d). Along with Col1A1, a significant decrease of FN1 in viable tumor regions was detected with VEGF overexpression. Representative viable and necrotic areas of immunostained sections from 231_WT tumors in Figure 4a and 231_VEGF tumors in Figure 4b show the decreased FN1 immunostaining in viable tumor regions of VEGF overexpressing tumors, but not in necrotic tumor regions. These data, summarized in Figure 4c,d, demonstrate the significant decrease of FN1 with VEGF overexpression in viable tumor regions (Figure 4c), but not in necrotic tumor regions with VEGF overexpression (Figure 4d).

**Figure 5.**
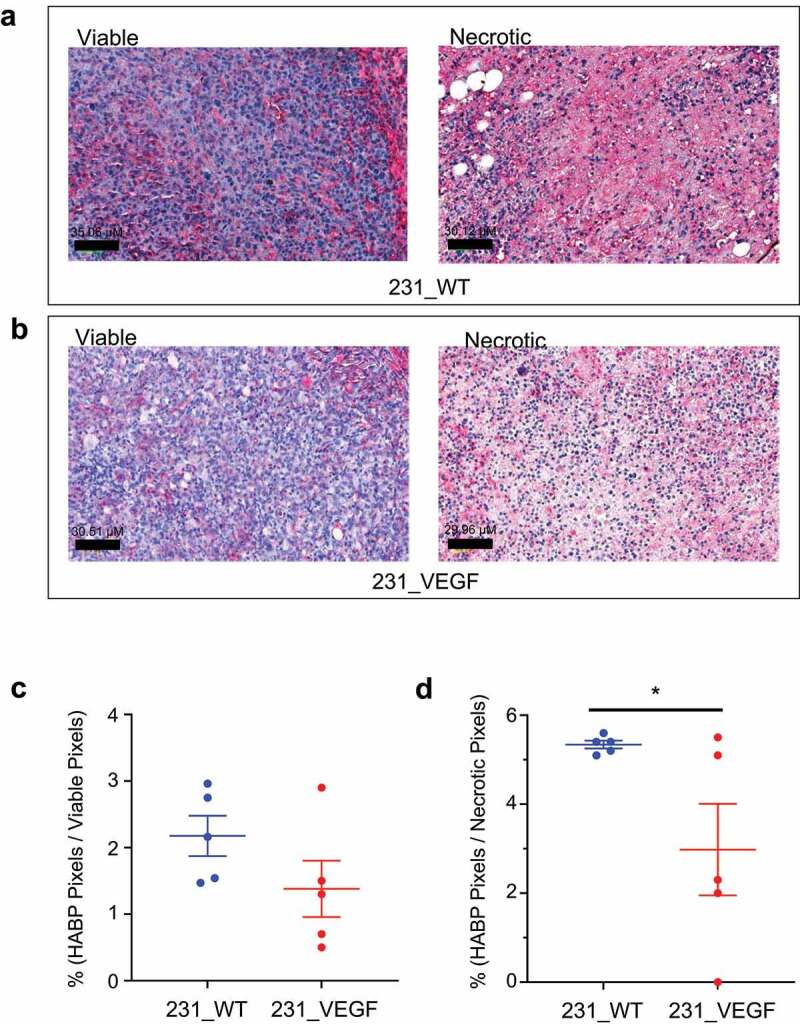
HABP Immunostaining. (a) Representative images of HABP immunostained tumor sections from viable (left) and necrotic (right) regions of 231_WT tumors. (b) Representative images of HABP immunostained tumor sections from viable (left) and necrotic (right) regions of 231_VEGF tumors. Quantification of HABP positive pixels normalized to the total pixel area of (c) viable tumor regions and (d) necrotic tumor regions from 231_WT (N = 5) and 231_VEGF (N = 5) tumors, 1 section analyzed per tumor. Values represent Mean ± S.E.M. *P ≤ .05.

Immunostaining of HA binding protein (HABP) was used to characterize changes in HA in VEGF overexpressing tumors. Representative viable and necrotic areas of HABP immunostained sections from 231_WT tumors in Figure 5a and 231_VEGF tumors in Figure 5b show decreased HABP immunostaining in viable tumor regions of VEGF overexpressing tumors, and in necrotic tumor regions. These data, summarized in Figure 5c,d, identified a trend (P ≤ .08) toward decreased HABP with VEGF overexpression in viable tumor regions (Figure 5c), and a significant decrease with VEGF overexpression in necrotic tumor regions (Figure 5d).

### Immunoblot analyses of tumor tissue and cells

**Figure 6.**
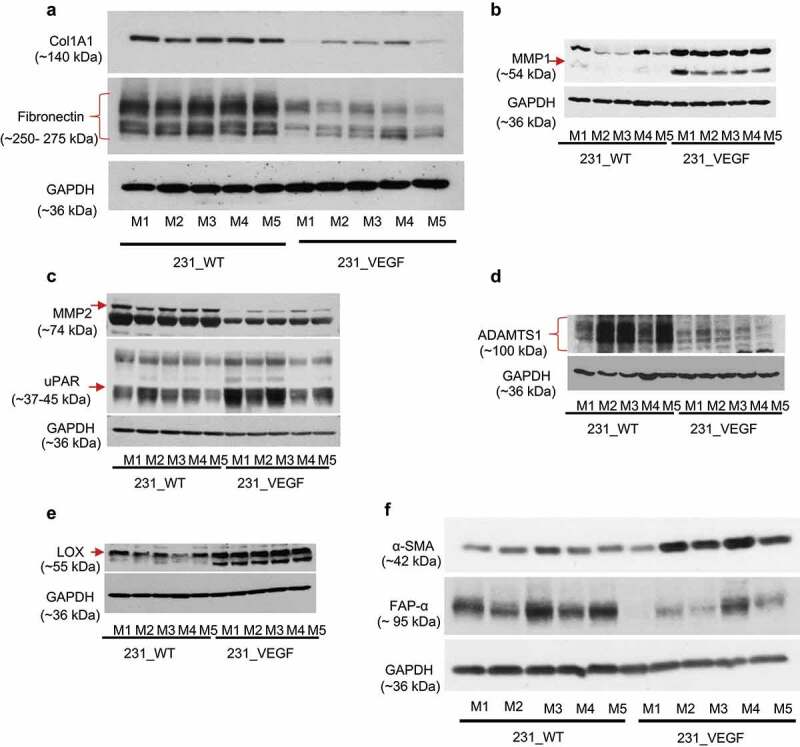
Immunoblot analysis of ECM proteins and enzymes in tumors. (a) Decrease of Col1A1 and fibronectin identified in 231_VEGF compared with 231_WT tumor samples. (b) Increase of MMP-1 in 231_VEGF compared with 231_WT tumor samples. (c) Decrease of MMP-2, and increase of uPAR in 231_VEGF compared with 231_WT tumor samples. (d) Decrease of ADAMST1 and (e) increase of LOX in 231_VEGF compared with 231_WT tumor samples. (f) Increase of α-SMA and decrease of FAP-α in 231_VEGF compared with 231_WT tumor samples. GAPDH was used as a loading control. n = 5 for 231_WT and n = 5 for 231_VEGF in all immunoblots.

Immunoblot analysis of tumors further confirmed a clear reduction of Col1A1 and FN1 protein with VEGF overexpression, as shown in Figure 6a and summarized in Supplementary Figure 2a.

Because ECM remodeling is frequently achieved by the action of various enzymes, we interrogated changes in MMP1, MMP2, MMP14, urokinase-type plasminogen activator receptor (uPAR), ADAM Metallopeptidase with Thrombospondin Type 1 Motif 1 (ADAMTS1), and LOX in VEGF overexpressing tumors. MMP1, uPAR and LOX clearly increased with VEGF overexpression as shown in Figures 6b-e and summarized in Supplemental Figures 2B-E. MMP2 and ADAMTS1 decreased with VEGF overexpression as shown in Figure 6c,d, and summarized in Supplementary Figures 2C and D. Unlike the other ECM degrading enzymes, we did not identify a clear change in MMP14 with VEGF overexpression (data not shown). Since CAFs play a major role in ECM synthesis, we also evaluated two well-established markers of CAFs, α-SMA and FAP-α. We identified a trend (P ≤ .08) of an increase of α-SMA, and a decrease of FAP-α in 231_VEGF tumors compared with 231_WT tumors (figure 6f, and Supplementary Figure 2 F). Multiple bands appearing in some immunoblots were either due to non-specific binding or due to protein phosphorylation.

**Figure 7.**
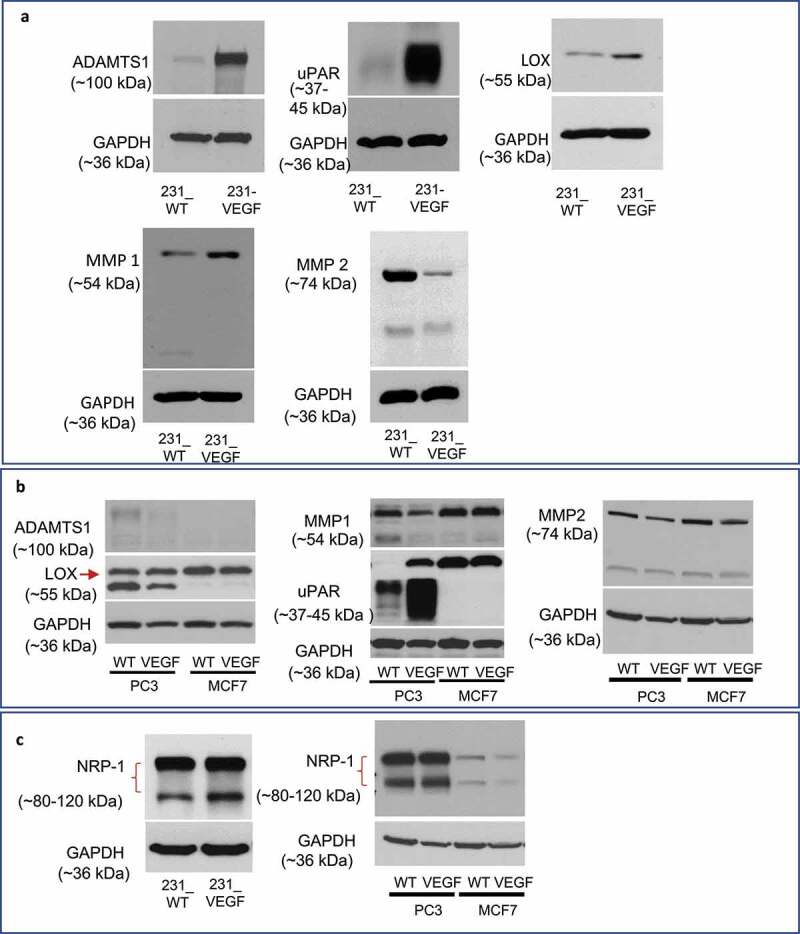
Immunoblot analysis of enzymes and receptors in cancer cells. (a) Changes in ADAMTS1, uPAR, LOX, MMP1 and MMP2 in 231_VEGF cells compared to 231_WT cells. (b) Changes in ADAMTS1, LOX, uPAR and MMP1 in wild type and VEGF overexpressing PC-3 and MCF-7 cells. (c) Changes in NRP-1 in MDA-MB-231, PC-3 and MCF-7 wild type and VEGF overexpressing cells.

We also characterized the same ECM remodeling enzymes in MDA-MB-231, PC-3 and MCF-7 VEGF overexpressing cells as shown in Figure 7. With the exception of ADAMTS1 that increased, MDA-MB-231 cells overexpressing VEGF showed changes similar to those observed in tumors for uPAR, MMP1, MMP2 and a trend toward increased LOX expression (P ≤ .09)(Figure 7a, Supplementary Figure 3A). Similar to MDA-MB-231 cells, PC-3 cells overexpressing VEFG showed an increase of uPAR, but ADAMTS1, LOX and MMP1 decreased or remained unchanged; MCF-7 cells did not exhibit any change in these enzymes with VEGF overexpression (Figure 7b). To understand the autocrine mechanisms causing these changes, we characterized the VEGF binding receptors VEGFR1 and NRP-1 in these cells. The data presented in Figure 7c demonstrate that both MDA-MB-231, and PC-3, but not MCF-7, wild-type cells expressed high levels of NRP-1 supporting the possibility of autocrine signaling. Similar levels of NRP-1 were detected in MDA-MB-231 (Figure 7c, Supplementary Figure 3B) and PC-3 VEGF overexpressing cells (Figure 7c). NRP-1 was low in MCF-7 VEGF overexpressing cells. VEGFR1 was not detected in wild type or VEGF overexpressing cells in all three cell lines (data not shown).

### mRNA changes in human samples and xenografts

**Figure 8.**
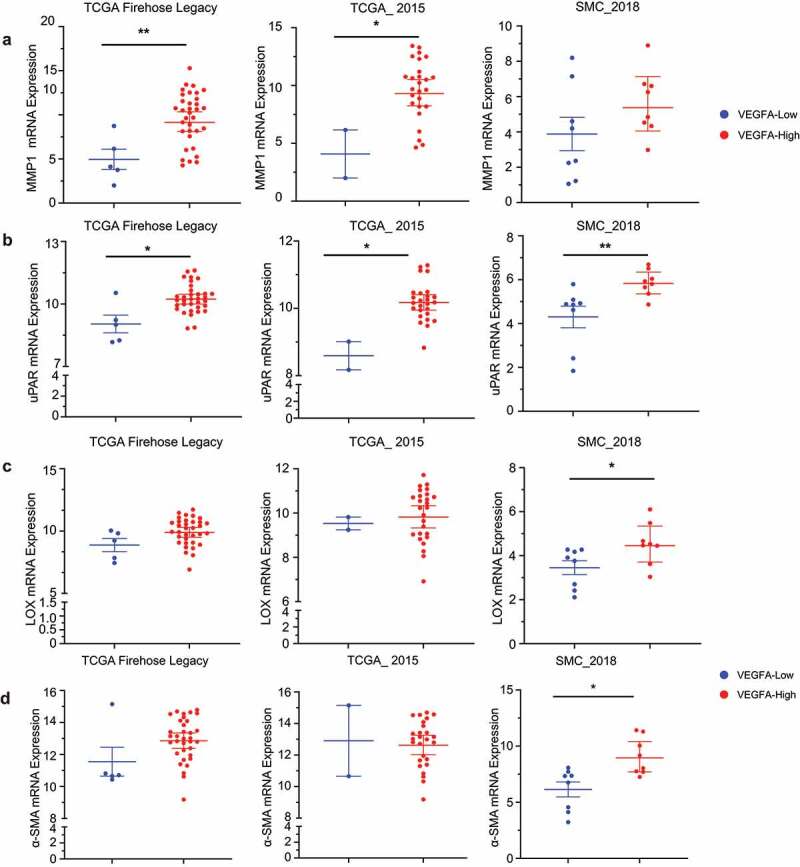
mRNA expression in treatment naïve TNBC with high and low VEGF expression. Comparison of RNA sequencing data expressed as log2 based on RSEM method of analysis of (a) MMP1, (b) uPAR, (c) LOX and (d) α-SMA in TNBC patient samples with high or low VEGFA expression from three different studies. Data represent geometric mean at 95% confidence interval from 5 low VEGFA (blue dots) and 34 high VEGFA (red dots) samples for the TCGA Firehose Legacy study, 8 samples each for low VEGFA and high VEGFA samples in the SMC 2018 Study. In TCGA_2015, there were only 2 samples in the low VEGFA (Blue) and 26 samples in the high VEGFA (red dots) group. Statistical analysis was performed with Mann Whitney test using GraphPad prism software and further confirmed with a two-tailed t-test. * P ≤ .05, ** P ≤ .005.

We mined the TCGA TARGET GTEx database to characterize mRNA changes in the degradative enzymes MMP1 (Figure 8a), uPAR (Figure 8b) and LOX (Figure 8c), and the CAF marker α-SMA (Figure 8d), in treatment naïve TNBC with high and low VEGF mRNA. Statistical analyses of the fold change were evaluated using the Mann-Whitney test and a 2-tailed t-test. Identical significant changes were identified with both tests. A significant increase of MMP1 mRNA in 2 of 3 studies, LOX mRNA in 1 of 3 studies with a trend toward increased expression in a second study (P ≤ .08 in TCGA Firehose Legacy), uPAR mRNA in all 3 studies, and α-SMA mRNA in 1 of 3 studies with a trend toward increased expression in the TCGA Firehose Legacy study (P ≤ .06) were identified in the high VEGF mRNA group compared to the low VEGF group, consistent with the changes observed in the tumor xenograft studies.

We characterized mRNA of the ECM proteins and degradative enzymes to understand transcriptional changes induced by VEGF overexpression in the xenografts. mRNA levels of Col1A1, Col1A2 and FN1 significantly decreased in 231_VEGF tumors (Supplementary Figures 4A-C) consistent with the reduction identified with IHC and in the immunoblots. Also, consistent with the immunoblots, mRNA of MMP1 and uPAR significantly increased (Supplementary Figures 4D-E), and mRNA of MMP2, ADAMTS1, and FAP-α significantly decreased (Supplemental Figures 4 F-H). We also confirmed a significant increase of VEGFA mRNA (Supplemental Figure 4I).

## Discussion

Our studies identified a clear reduction of key ECM components, Col1A1, FN1 and HA in MDA-MB-231 xenografts with VEGF overexpression. The patterns of Col1 fibers, identified by Haralick feature analysis, were also altered by VEGF overexpression. Increased expression of VEGF was confirmed directly in cells and tumors, as well as from the functional changes of increased vascularity detected by the endothelial cell marker, CD31, and increased tumor growth. Changes in enzymes such as an increase of MMP1, uPAR, and LOX, and a decrease of MMP2 and ADAMTS1, together with an increase of CAFs, most likely contributed to the ECM changes in the VEGF overexpressing tumors. Increases in mRNA expression for various matrix degrading enzymes together with the increase in α-SMA mRNA in the xenografts were confirmed in the TCGA analysis of treatment naïve TNBC with high and low VEGFA data sets. Our purpose with the TCGA analysis was to determine if the protein expression changes identified in our tumor models were reflected in publicly available human data, although the probed genes in the TCGA data may not necessarily predict protein expression.

The autocrine and intracrine roles of VEGF^34^ were evident from the changes in enzymes observed in VEGF overexpressing cancer cells. With the exception of ADAMTS1 that decreased in 231-VEGF tumors but increased in cells, the increase of uPAR, LOX, MMP1 and a decrease of MMP2 observed in the tumors was also observed in 231_VEGF cells that suggested that the enzymatic changes driving the ECM changes occurred directly within the cancer cells.

uPAR increased significantly in PC-3 prostate cancer cells overexpressing VEGF, and was consistently higher in the human TNBC data. MCF-7 breast cancer cells that are ER +ve and poorly invasive did not show any alterations of the degradative enzymes investigated.

The autocrine changes mediated by VEGF most likely occurred through binding of VEGF to NRP-1 that showed high expression in the MDA-MB-231 and PC-3 wild type and VEGF overexpressing cells, but not MCF-7 cells that was consistent with the absence of any alterations of the degradative enzymes investigated following VEGF overexpression. Previous observations of an increase of ECM degradation by VEGF overexpressing breast cancer cells^9^ are also consistent with enzyme changes occurring through autocrine signaling in the cells. Similar autocrine signaling mediated modulation of enzymes by VEGF was previously observed in A549 lung cancer cells,^35^ vascular smooth muscle cells,^23^ and chondrocytes.^36^ Both paracrine and autocrine signaling play a role in tumor microenvironment changes mediated by VEGF as demonstrated in a study where suppressing VEGF decreased metastasis *via* disrupting both the autocrine and paracrine signaling loops of VEGF.^37^

In MDA-MB-231 tumors, the three enzymes that increased with VEGF overexpression were MMP1, uPAR and LOX, while MMP2 and ADAMTS1 decreased. The increase of uPAR is consistent with the reduction of the ECM components observed here since uPAR plays a role in generating plasmin and activating the MMPs. MMP1, or collagenase 1, is a matrix metalloproteinase that specifically degrades collagen 1. The increase of MMP1 can explain the reduction of Col1 fibers observed with VEGF overexpression, despite the increase of LOX the enzyme that plays a role in cross-linking Col1 fibers.^38^

The reduction of MMP2 and ADAMTS1 may have indirectly contributed to ECM changes. MMP2 is a type IV collagenase that plays a role in releasing growth factors bound to the ECM and in degrading the basement membrane.^39^ ADAMTS1 is part of a family of extracellular proteolytic enzymes known to have diverse functions related to ECM remodeling, angiogenesis, cell migration and organogenesis. Dysregulation of these enzymes has been implicated in various diseases including multiple cancers. In breast cancer, overexpression of ADAMTS1 was shown to promote tumor progression and to be upregulated in metastatic TNBC (reviewed in^40^). Under physiological conditions, ADAMTS1 has been shown to sequester VEGF165 thereby acting as an angiogenesis inhibitor; binding of ADAMTS1 to VEGF165 disrupts the binding and phosphorylation of VEGFR2 leading to the suppression of endothelial cell proliferation.^41,42^

In wound-healing, VEGF-mediated increases of collagen and FN1 have been frequently documented.^43^ During wound-healing, the increased vascular permeability induced by VEGF results in extravasation of proteins such as fibrin and FN1 which provides a temporary matrix to nourish fibroblasts, facilitate their motility, and induce them to deposit a more structured collagen stroma.^44^ Our data revealed that, unlike during wound repair, in our TNBC xenograft model, VEGFA overexpression resulted in a decrease of Col1A1 and FN1, despite an increase of the CAF marker α-SMA, indicating that VEGF-mediated changes in the ECM are reprogrammed in cancer. Although, to the best of our knowledge, a direct evaluation of ECM changes with VEGF overexpression has not been performed, changes in the tumor ECM observed with anti-VEGF treatments provide useful insights in understanding the changes observed.

Three major ECM proteins, Col1A1, FN1 and HA, significantly decreased with VEGF overexpression. Collagen fibers are the most abundant structural protein in the ECM. TNBC, in particular, has a significantly increased deposition of collagen as well as increased matrix stiffness compared with luminal breast cancer subtypes.^45^ Previous studies have identified an association between increased Col1 fibers and metastasis in breast^46^ and prostate cancer.^47^ Here, SHG microscopy together with immunohistochemistry and molecular analysis clearly demonstrated that VEGF overexpression reduced Col1 fibers, protein and mRNA. Hypoxia is a potent transcriptional regulator of VEGF. The changes in Col1 fibers with VEGF overexpression are consistent with earlier observations that hypoxic tumor regions exhibit fewer Col1 fibers.^26^

Similar to Col1, FN1 was reduced by VEGF overexpression. FN1 is a glycoprotein that is present in dimeric and multimeric form in the ECM. It binds through an RGD sequence to endothelial cells, and is frequently found localized with endothelial cells.^48^ Here, despite an increase of the endothelial cell marker, CD31, with VEGF overexpression, FN1, in viable tumor regions, significantly decreased. FN1 plays important roles in cell migration, growth, and differentiation.^49^ FN1 interacts with Col1 in the tumor ECM during tumorigenesis.^50^ FN1 has been shown to contribute to tumor growth, progression, migration, and response to therapy (reviewed in^51^). In breast cancer, intracellular FN1 has been associated with increased metastasis.^52^ Treatment of a colon cancer xenograft with bevacizumab resulted in a significant increase of fibronectin,^53^ indirectly supporting our observation that increased VEGF reduces fibronectin.

Since Col1 and FN1 are associated with increased metastasis,^46,52,54^ their reduction with VEGF overexpression, which is also known to increase metastasis in these tumors,^9^ was surprising. It is possible that a less dense ECM may be more conducive to metastasis, or that although VEGF overexpression results in an ECM that is less permissive to metastasis, increased vascularity, and invasiveness override this with the net outcome of an increase of metastasis.

In addition to Col1 and FN1, we observed a reduction of HA, a high-molecular weight, unbranched, nonsulfated glycosaminoglycan that is an important structural component of various tissues including the tumor ECM. HA regulates adhesion, cell proliferation, EMT, gene expression, invasion, motility, signaling, metastasis, and morphogenesis^55,56^ by its ability to bind to various HA binding proteins collectively called hyaladherins.^57,58^ Additionally, breakdown products of HA can stimulate angiogenesis.^59^ Although an increase of HA is frequently associated with increased invasion,^60^ degradation of HA has also been observed in many pathologies including inflammation and cancer.^61^ Studies of colorectal cancer liver metastases treated with anti-VEGF therapy have identified an increase of HA and sulfated glycosaminoglycans and increased tumor stiffness after anti-VEGF treatment, although no significant changes in collagen were detected.^62^

The ECM changes observed here represent a net outcome of synthesis by CAFs and degradation by enzymes. CAFs are one of the most abundant stromal cell population in the tumor microenvironment, performing diverse functions such as synthesis and remodeling of the ECM, promoting migration, invasion, and metastasis, and affecting immune cell function.^63,64^ CAFs are a heterogeneous population of cells derived from multiple cellular sources.^63^ Here, α-SMA, which is expressed by most CAFs,^65^ increased with VEGF overexpression. These results are consistent with an earlier study in which α-SMA levels increased in VEGF overexpressing TNBC.^8^ However, FAP-α, a marker of a subset of CAFs associated with immune suppression decreased.^66^ It is possible that different CAF subsets may have different responses to VEGF, and that the ECM synthesis capabilities of subsets of CAFs may be different. In a murine intrahepatic cholangiocarcinoma model, blocking placental growth factor (PIGF)-α, a member of the VEGF family, impacted only one subset of CAFs that expressed low levels of Col1.^67^ Reduction of FAP-α expressing CAFs may have contributed to the changes in the ECM observed here.

Our data highlight the autocrine role of VEGF in increasing uPAR and other degradative enzymes and support the use of disrupting these autocrine or intracrine loops for treatment. A limitation of our study is that we used a single time point as a snap-shot to evaluate changes in the ECM, CAFs and enzymes in these tumors. The availability of noninvasive imaging to image the ECM, CAFs, and enzymes will allow evaluation of the spatial and temporal evolution of the reprogramming of the ECM and its causes that occurs with VEGF overexpression in tumors, compared to healing wounds.^44^ Such studies may provide additional insights into the role of VEGF in tumor progression and metastasis.

## Supplementary Material

Supplemental Material

## Data Availability

The data that support the findings of this study are available from the corresponding author, Zaver Bhujwalla, upon reasonable request.
