# Supplementary material for: Reprogramming of VEGF-mediated extracellular matrix changes through autocrine signaling
Source: Cancer Biol Ther. 2023 Mar 9;24(1):2184145. doi: 10.1080/15384047.2023.2184145 (PMC10012930; doi:10.1080/15384047.2023.2184145)
Supplement: Supplemental Material [file KCBT_A_2184145_SM4047.zip › Supplementary Figure Legends.docx]

**Supplementary Figure Legends**

**Supplementary Figure 1: VEGF mRNA expression.** (A) Fold change of VEGF mRNA in PC-3_VEGF overexpressing cells (n=3) compared to PC3_WT cells (n=3) (left panel). Validation of the overexpression of VEGF in the supernatant of PC3_VEGF cells (n=3) compared to PC_WT cells (n=3) and in the cell lysate (right panel) of PC3_VEGF and PC3_WT cells (n=3 per group) by ELISA (middle and right panel. Values represent Mean ± S.E.M. ****P≤ 0.00005.

(B) Fold change of VEGF mRNA in MCF-7_VEGF overexpressing cells (n=3) compared to MCF-7_WT cells (n=3) (left panel). Validation of the overexpression of VEGF in the supernatant of PC3_VEGF cells (n=3) compared to PC_WT cells (n=3), and in the cell lysate of PC3_VEGF and PC3_WT cells (n=3 per group) by ELISA (middle and right panel). Data represent geometric mean at 95% confidence interval + SEM from three biological experiments for mRNA expression and from three biological experiments for ELISA. Statistical analysis was performed on the fold changes. ** P < 0.005, **** P < 0.00005.

**Supplementary Figure 2:** Quantification of band intensity of immunoblots from *in vivo* samples. Densitometry of Col1A1 and FN1 (A), MMP1 (B), MMP2 and uPAR (C), ADAMTS1 (D), LOX (E) and α-SMA and FAP-α (F) protein expression relative to GAPDH expression in 231_WT (n=5) and 231_VEGF (n=5). Statistical analysis was performed on the ratio of band intensities of various protein of interest to that of GAPDH band intensity. Data represent Mean + SEM. *P < 0.05, ** P< 0.005.

**Supplementary Figure 3:** Quantification of band intensities of immunoblots from cell lysates. (A) Densitometry of ADAMTS1, uPAR, LOX, uPAR, MMP1 and MMP2 intensity relative to GAPDH in 231_WT (n=3) and 231_VEGF (n=3). (B) Densitometry of NRP1 expression in 231_WT (n=3). Data represent Mean + SEM. *P < 0.05, ** P< 0.005.

**Supplementary Figure 4:** **mRNA expression in xenografts.** Fold change in mRNA expression of (A) Col1A1, (B) Col1A2, (C) FN1, (D) MMP1, (E) uPAR, (F) MMP2, (G) ADAMST1, (H) FAP-α, and (I) VEGF expression in 231_WT (n=10) and 231_VEGF (n=10) tumors. Statistical analysis was performed on the fold change. Values represent Mean + SEM. *P < 0.05, ** P< 0.005, **** P

< 0.00005.
