# Supplementary figures and images for: Reprogramming of VEGF-mediated extracellular matrix changes through autocrine signaling
Source: Cancer Biol Ther. 2023 Mar 9;24(1):2184145. doi: 10.1080/15384047.2023.2184145 (PMC10012930; doi:10.1080/15384047.2023.2184145)

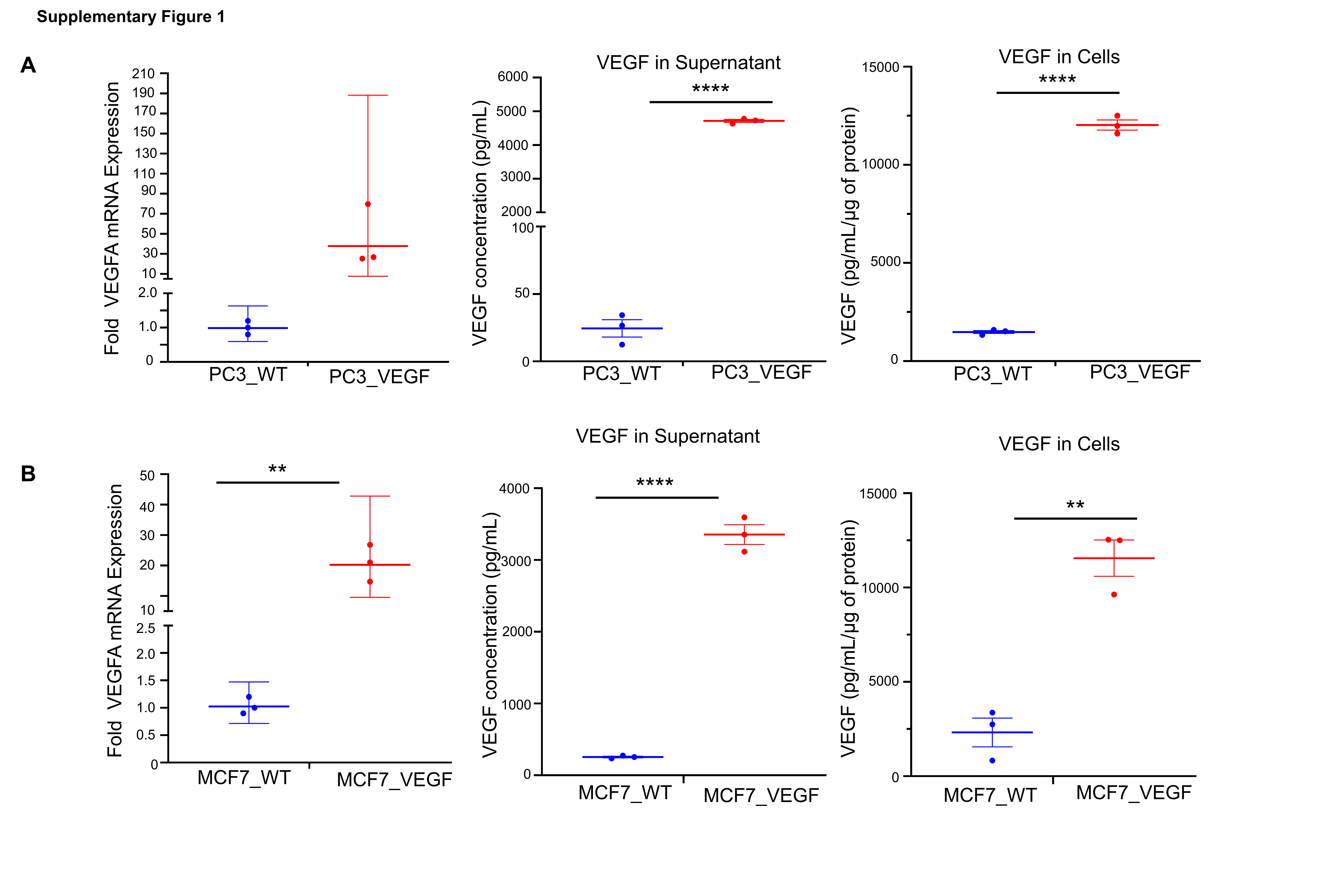

Supplement: Supplemental Material [file KCBT_A_2184145_SM4047.zip › Goggins_et_al_Second_Revised_Supp_Figure 1.TIF]

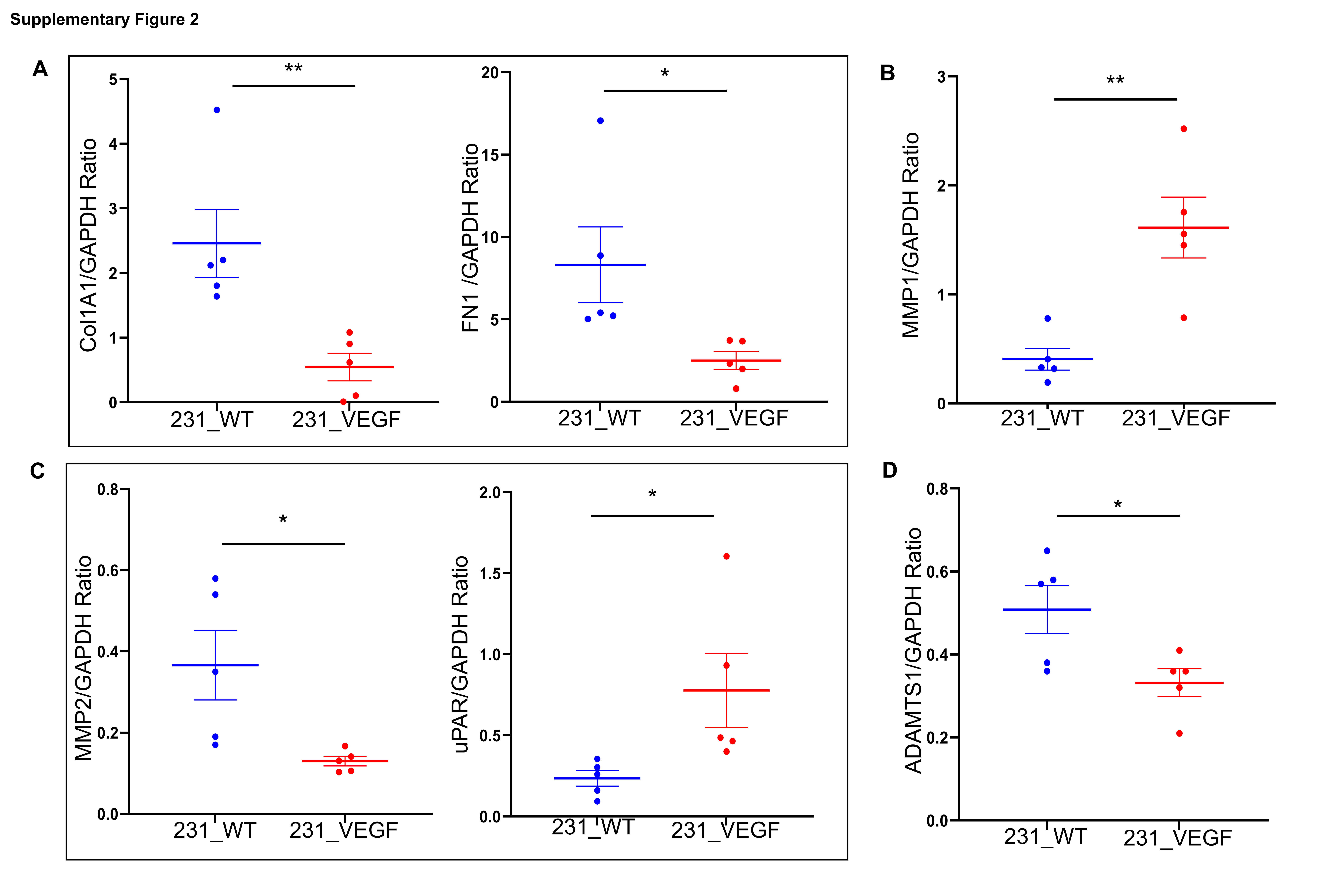

Supplement: Supplemental Material [file KCBT_A_2184145_SM4047.zip › Goggins_et_al_Second_Revised_Supp_Figure 2.TIF]

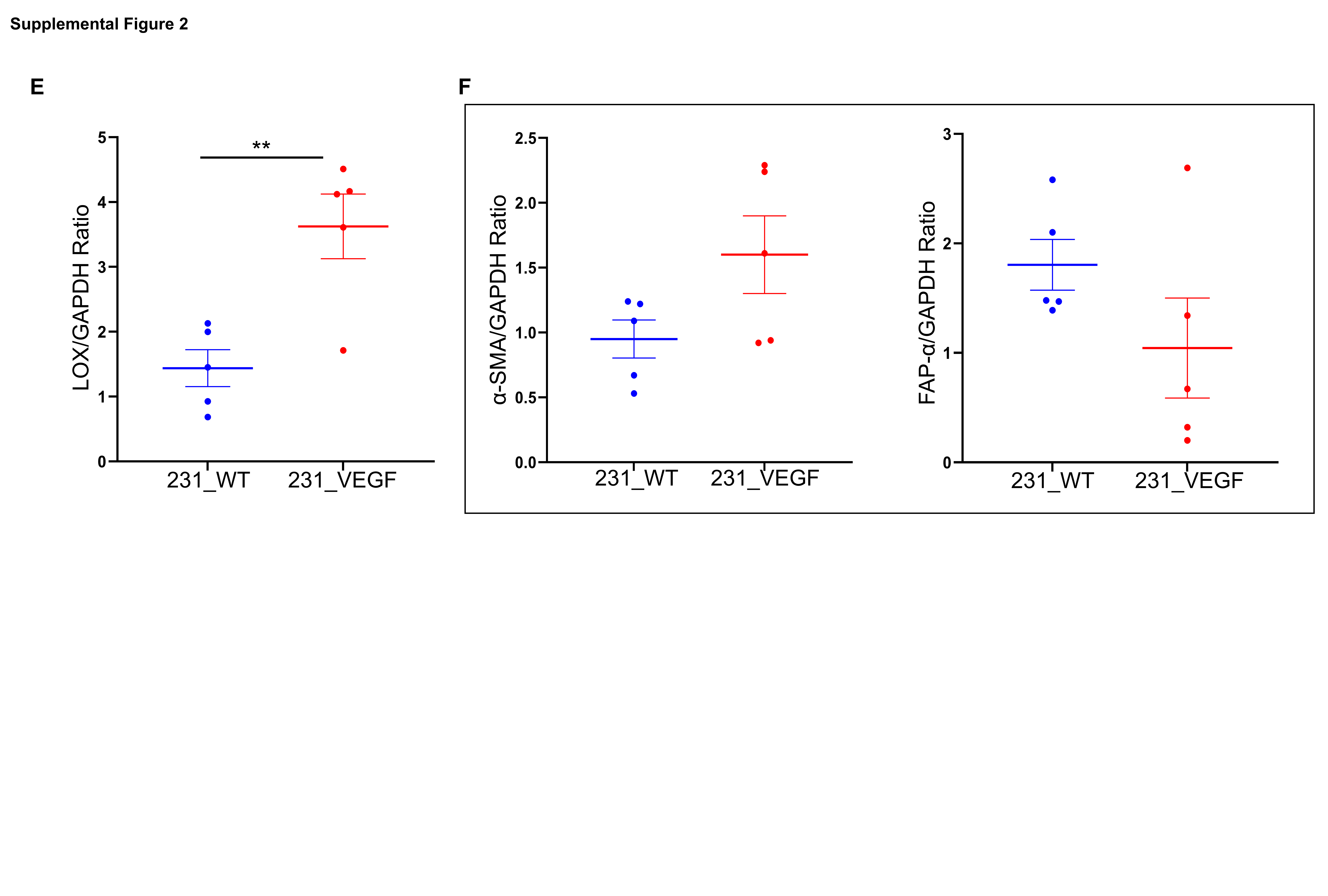

Supplement: Supplemental Material [file KCBT_A_2184145_SM4047.zip › Goggins_et_al_Second_Revised_Supp_Figure 2B.TIF]

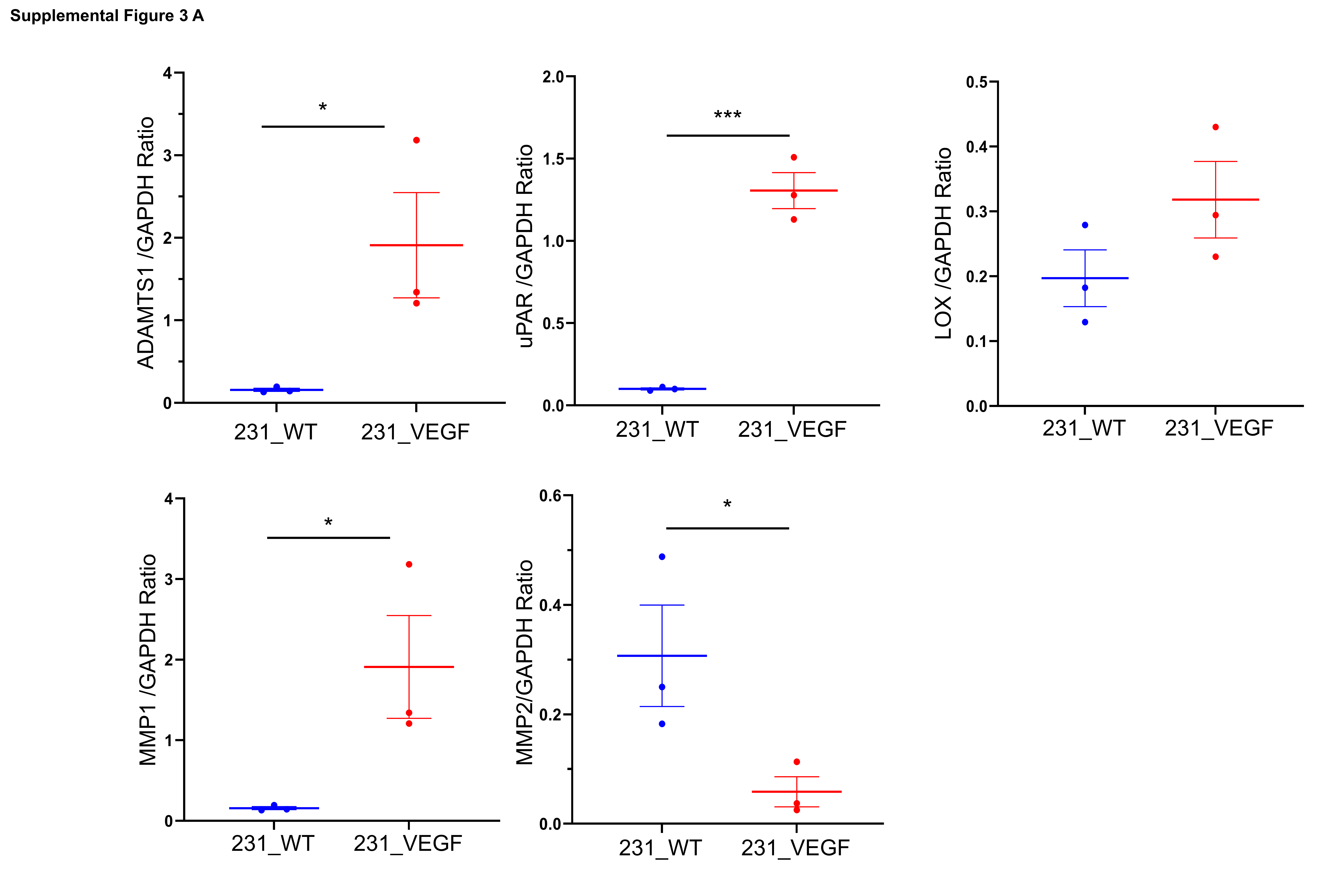

Supplement: Supplemental Material [file KCBT_A_2184145_SM4047.zip › Goggins_et_al_Second_Revised_Supp_Figure 3.TIF]

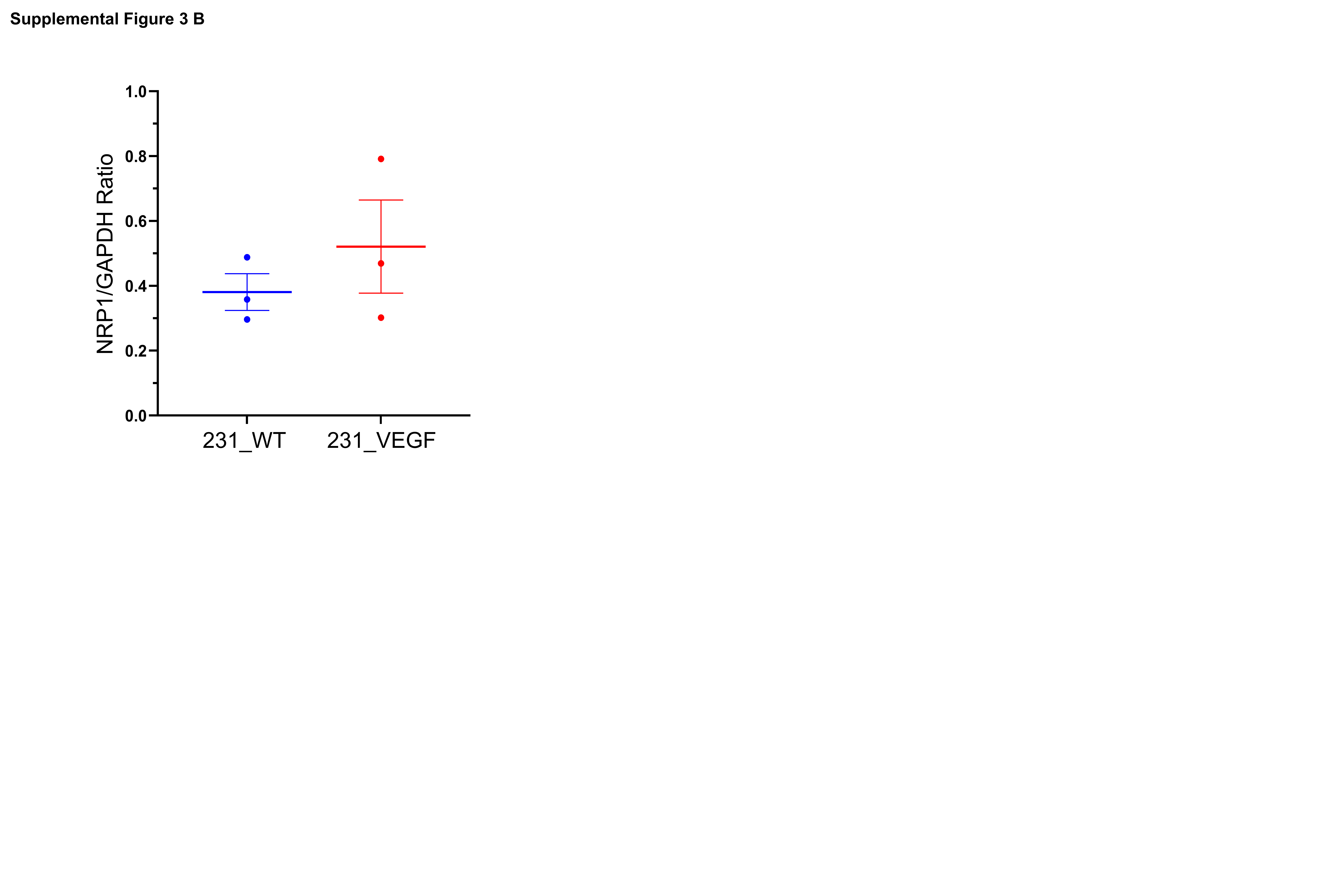

Supplement: Supplemental Material [file KCBT_A_2184145_SM4047.zip › Goggins_et_al_Second_Revised_Supp_Figure 3B.TIF]

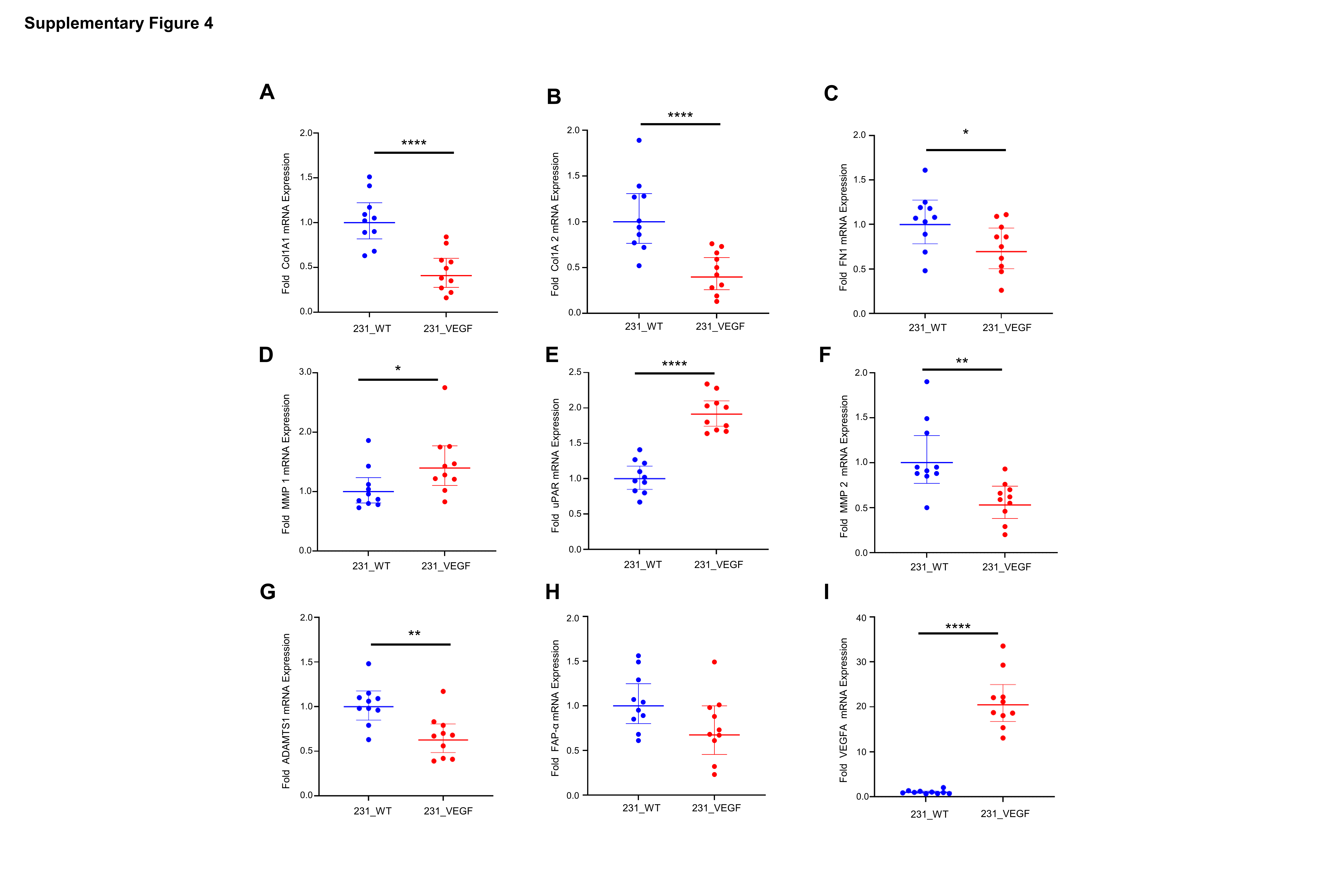

Supplement: Supplemental Material [file KCBT_A_2184145_SM4047.zip › Goggins_et_al_Second_Revised_Supp_Figure 4.TIF]
